# Sensitivity, reliability and construct validity of GPS and accelerometers for quantifying peak periods of rugby competition

**DOI:** 10.1371/journal.pone.0236024

**Published:** 2020-07-20

**Authors:** Samuel T. Howe, Robert J. Aughey, William G. Hopkins, Bryce P. Cavanagh, Andrew M. Stewart

**Affiliations:** 1 Institute for Health and Sport, Victoria University, Melbourne, Australia; 2 Melbourne Rebels Rugby Union Club, Melbourne, Australia; 3 Defence Institute, Oslo, Norway; 4 Shandong Sport University, Jinan, China; National Taipei University of Nursing and Health Sciences, TAIWAN

## Abstract

Training prescription and monitoring of team-sport athletes rely on accurate quantification of player movement. Our aim was to determine the sensitivity, reliability and construct validity of measures derived from a wearable device incorporating Global Positioning System (GPS) and accelerometer technology to quantify the peak periods of rugby competition. Match movement data were collected from 30 elite and 30 sub-elite rugby union players across respective competitive seasons. Accelerometer and GPS measures were analysed using a rolling average to identify peak movement for epochs ranging from 5 to 600 seconds. General linear mixed modelling was used to quantify the effects of playing position and match-half on the peak movement and variabilities within and between players represented reliability of each measure. Mean positional differences and match-half changes were assessed via standardisation and magnitude-based decisions. Sensitivity of measures was quantified via evaluation of ("signal") and typical error of measurement ("noise"). GPS and accelerometer measures had poor sensitivity for quantifying peak movement across all epochs and both levels of rugby union competition (noise 4× to 5× the signal). All measures displayed correspondingly low reliability across most epochs and both levels of competition (ICC<0.50). Construct validity was evident in mean differences between playing positions and match halves that were consistent with expected activity profiles in rugby union. However, it was clear from the pattern of differences across epoch durations and levels of competition that GPS and accelerometer measures provided different information about player movement. The poor sensitivity and low reliability of GPS and accelerometer measures of peak movement imply that rugby union players need to be monitored across many matches to obtain adequate precision for assessing individuals. Although all measures displayed construct validity, accelerometers provided meaningful information additional to that of GPS. We recommend using accelerometers alongside GPS to monitor and prescribe match respresentative training.

## Introduction

Using wearable global positioning systems (GPS) and inertial sensors to quantify athletic movement is an application of the technology long preceded by navigation and military applications [1]. Quantification of athletic movement via wearable technology is important for sporting practitioners as it provides objective data to inform the decision-making process around training load management [2], training prescription [3], player readiness to play [4], injury risk [5], and player interchange decisions [6, 7]. The prolific adoption of GPS with integrated inertial sensor technology in elite team sports is testament to its perceived worth and impact on player and team preparation and performance.

Position, velocity and distance can be derived via GPS [8]. Subsequently, change in velocity (acceleration and deceleration) may be calculated [9] and potentially used in combination with velocity-based events to estimate the energy cost of exercise (metabolic power) [10]. Whilst GPS athlete tracking data can be of great value to practitioners, it has reduced validity and reliability for quantifying rapid changes of direction [11] and velocity [12, 13], estimating metabolic power [14] and for assessing short duration, high-velocity tasks that frequently occur in team sports [13, 15]. Movements that incur little horizontal displacement (e.g., collisions, tackles and many sport-specific movements) are also likely to be underestimated by GPS [16]. Further, positional and match-half differences in athlete maximal movement were underestimated by GPS technology when compared to accelerometers during professional rugby union match-play [17]. In light of these findings, authors recommended that researchers and practitioners use accelerometers alongside GPS technology to adequately quantify important positional differences and match-half changes in athlete movement during collision-based team sports [17].

Manufacturers of accelerometer technology used by sporting practitioners and scientists have created modified vector magnitude proprietary algorithms, with frequently published measures being PlayerLoad^™^ (Catapult Sports) [16] and BodyLoad^™^ (GPSports) [18]. Vector magnitudes sum the squared instantaneous rate of change in acceleration in three orthogonal axes accumulated over time, providing an estimate of totality of movement often referred to as external load. Accelerometers have been used to quantify athlete external load [16] and energy expenditure [19] during training and competition, with PlayerLoad^™^ moderating the recovery response of footballers [20]. Accelerometers are reliable in laboratory [21] and field settings [22] and can accurately detect individual contact events [23], sport-specific movements [24], and alterations in movement strategies, efficiency or kinematic changes [4, 25]. Unlike GPS, accelerometers can also operate within indoor environments, providing greater utility [26]. Accelerometers provide valuable additive external load information to GPS that may aid practitioners in more accurately quantifying player totality of movement in collision-based team sports [17]. Quantifying external load during collision-based team sports may help practitioners to prescribe and monitor training in a more objective manner, carefully balancing the need for physiological and biomechanical load to induce positive adaptations whilst mitigating overuse to reduce injury likelihood, or put more simply, balance fitness and fatigue [27].

Team sports that contain a substantial collision-based component (e.g., rugby union, rugby league, National Football League and Australian Rules Football) are characterised by low-intensity activity interspersed with frequent bouts of high-intensity activity [28, 29]. Despite the majority of team-sport competition being spent at submaximal intensity, high-intensity activities are often aligned with key events that determine match outcome [30, 31], signifying the importance of physically conditioning athletes for these intense periods of match-play. The most intense or peak periods of football competition do not often fall completely within a pre-defined period of time and therefore these methods underestimate the most intense periods of match-play and overestimate subsequent periods of activity [32–34]. During elite soccer competition the peak periods of high-velocity running distance were identified using either pre-defined (distance covered in 5-minutes at every 5-minute time point) or rolling time periods (distance covered in 5 minutes from every time point). Rolling or moving average methods involve analysing raw instantaneous data from the device used. For example, GPS device data are commonly sampled at 10 Hz (i.e. ten times per second) and accelerometer data typically at 100 Hz (i.e. one hundred times per second). To identify the peak periods of competition using a moving average approach, one must select a duration of interest (e.g. 5 minutes), with that window of time then moved across every second of the competition, collecting a moving average from every single time point. For example, using a one-minute window that equates to 600 samples (60 s with ten samples per second using 10 Hz GPS), the moving average would be applied to a player’s match data file as follows: 0–600, 1–601 s, 2–602 s, 3–603 seconds etc., to identify the one-minute peak measure/s of interest [35]. During professional soccer competition, peak high-velocity running distance was underestimated by up to 25% using pre-defined time period analysis, with the subsequent period distances overestimated by up to 31% when compared to rolling time period analysis. When the distance decline in high-velocity running between the peak and following period were examined, there was up to a 52% greater reduction in running performance using rolling vs. pre-defined periods [32]. Likewise during international rugby competition, both high-speed running (>5 m.s^-1^) and relative distance (m.min^-1^) were consistently underestimated by pre-defined compared to rolling period analyses of 60–300 seconds [33]. Pre-defined epoch analyses on average underestimated relative distances covered by ~11% and high-speed running by up to ~20% compared to rolling epoch analyses, with the greatest underestimations occurring using the 60 second epoch (95% confidence interval, high-speed running: -6.05 to -4.70 m.min^-1^, relative distance: -18.45 to -16.43 m.min^-1^) [33]. Similarly in English Championship soccer matches, pre-defined epoch analyses of 60–600 seconds underestimated peak movement intensities of competition when compared to rolling epoch analyses for both total distance (∼7–10%) and high-speed (∼12–25%) distance, irrespective of playing position [34]. Therefore, it is recommended that researchers and practitioners use rolling/moving time period analyses when trying to accurately identify and quantify the peak periods of football competition [32].

Duration- and position-specific player movement differences have been observed during the most intense periods of match-play across various football codes including: rugby league [3], rugby union [36], Australian Rules Football [37] and soccer [38]. These investigations provided valuable insights into the highly intermittent nature of team-sport movement and highlighted that rolling time-motion analyses may assist practitioners in the design and prescription of training that is more representative and specific to competition. However, the sensitivity, reliability and construct validity of GPS- and accelerometer-derived measures for quantifying player movement during the most intense periods of match-play in team sports is not known, limiting a practitioner’s ability to interpret and use such data to inform practice. Our aim was therefore to determine the sensitivity, reliability and construct validity of measures derived from GPS and accelerometer technology to quantify the most intense periods of rugby union match-play.

## Materials and methods

### Participants

Movement data were collected via integrated GPS and accelerometer units for 60 professional rugby union players (30 elite and 30 sub-elite) across two team’s respective seasons. The 30 elite players (18 forwards and 12 backs) played in the 2015 Super 15 Rugby competition, an international rugby union competition played between 5 Australian, 5 New Zealand and 5 South African teams. The Super 15 competitive season comprised of 18 rounds with 2 bye rounds per team, making 16 total matches (8 home, 8 away). The 30 sub-elite players (16 forwards and 14 backs) played in the 2014 National Rugby Championship, an Australian competition played between 9 teams from 5 states and territories, with the season comprising of 8 matches (4 home, 4 away) prior to a finals series for the top 4 finishing teams. The National Rugby Championship is the highest standard of rugby union played in Australia below Super 15 and international representative rugby. The data set for sub-elite includes 7 regular season matches and the semi**-**final (8 matches total), as 1 match did not meet our inclusion criteria (see Methods, data filtering and processing section). Players were grouped by playing position into forwards and backs rather than more specific playing positions (e.g., prop, centre, scrum-half) to increase precision of estimates and to first assess if the respective technologies were sensitive enough to quantify broader positional classifications prior to comparing specific positional groupings. Elite and sub-elite participant numbers and physical characteristics can be seen in Table 1. All players gave informed consent to participate and the study was approved by the Victoria University Human Research Ethics Committee.

**Table 1:** Participant numbers and physical characteristics of the rugby union players in the elite (Super 15) and sub-elite (National Rugby Championship) professional teams.

	Elite	Sub-elite
Number of participants	30	30
Number of forwards	18	16
Number of backs	12	14
Mean age (y)	25 ± 4	24 ± 4
Mean height (cm)	187 ± 7	185 ± 7
Mean body mass (kg)	106 ± 12	106 ± 13
Forwards age (y)	25 ± 4	24 ± 3
Forwards height (cm)	187 ± 8	187 ± 8
Forwards body mass (kg)	113 ± 8	115 ± 10
Backs age (y)	25 ± 4	24 ± 4
Backs height (cm)	185 ± 5	183 ± 5
Backs body mass (kg)	95 ± 6	96 ± 9

### Equipment and data collection

Match movement data was collected via commercially available OptimEye^™^ S5 GPS and GLONASS-enabled units with an embedded tri-axial piezoelectric accelerometer (firmware version 7.22, Catapult Sports, Melbourne, Australia). Prior to data collection during match-play, the devices were turned on and left outside on the playing surface in an open area to attain a satellite connection before placing them on the rugby players. The devices (96 mm × 52 mm × 14 mm, weighing 67 g) were placed within a custom-made tightly-fitting pouch within the back of the player’s playing uniform, situated between their scapulae. To minimize inter-unit variability, each player was assigned the same unit for the entirety of the respective seasons [39].

Manufacturers of the technology used in the present study did not reveal to authors the specific model of accelerometer. However, we have attempted to provide high level specification descriptions of the technology used where possible. Sampling frequencies for the GPS and tri-axial accelerometer were 10 Hz and 100 Hz respectively, with the accelerometer having an output range of ±16 *g*. Each unit has its own microprocessor, gyroscopes (3D, 8000 deg·s^-1^, up to 1000 Hz), magnetometers (3D, 100 Hz, full scale of 1200 micro tesla), 2 GB internal flash memory, 250 m wireless frequency transmission, a high-speed USB interface to record, store and retrieve data, a lithium ion rechargeable battery with 6-hours life and is water resistant. Accelerometer signal drift errors from the baseline gravity measure are negligible with temperature changes from 15 to 35°C, using the predessor model accelerometer (Catapult MinimaxX 2.0, Kionix: KXP94) to that used in the present study [22]. Positioning the accelerometer between player’s scapulae near the surface of the skin during a whole-body dynamic task such as rugby union should not exceed the manufacturer specified operating temperature ranges of -40 to 85°C.

### Measures of maximum mean movement

The three measures of maximum mean or peak movement investigated were: Playerload^™^ (au, accelerometer-derived), mean speed (m.min^-1^, GPS-derived) and metabolic power (W.kg^-1^, GPS-derived) as they provide estimates of global external load and are frequently reported in research and used in practice. Acceleration, total distance, high-speed running distance and estimated metabolic power were ranked as the most important variables in the eyes of elite football practitioners [40], lending further support for the chosen measures.

PlayerLoad^™^ is a Catapult Sports proprietary vector magnitude, mathematically expressed as the square root of the sum of the squared changes in acceleration in three orthogonal planes over the sampling interval (set at 100 Hz):

$${\text{PlayerLoad}}^{™}=\surd \left(\Delta {\text{Forward}}^{2}+\Delta {\text{Side}}^{2}+\Delta {\text{Up}}^{2}\right),$$

where Forward, Side and Up refer to directions of acceleration, and Δ refers to the change over the sampling interval (10 ms) [22].

The units of acceleration are m.s^-2^, but the Catapult software applies an arbitrary unknown scaling factor when this measure is accumulated and may have applied such a factor to this "instantaneous" measure. We have therefore shown its units as arbitrary (au).

Metabolic power is a GPS-derived measure of power that considers the energetic cost of accelerated running on flat terrain to be energetically analogous to running on an equivalent uphill slope at a constant speed [10]. Instantaneous metabolic power output (W.kg^-1^) of an individual may subsequently be calculated if acceleration and velocity is known [10, 41]. Whilst the measure was lauded and widely applied when first published, extensive validity and reliability testing had not yet been carried out. The application of metabolic power in team sports has been questioned in recent years [14, 42], yet its perceived importance and application is still prevalent [40]. We were therefore interested to investigate whether metabolic power has merit for quantifying external load during the most intense periods of a collision-based team sport like rugby union.

Mean speed or relative distance (m.min^-1^) is a GPS-derived measure that expresses the absolute distance an athlete covers relative to the time they spent on the field. For instance, if an athlete covers 8000 m during an 80-minute rugby union match, their relative distance would be 100 m.min^-1^. Quantification of mean speed or relative distance may assist subsequent training prescription and monitoring of intensity and enables reasonable comparison between full-match and substitution players and between different sporting codes [26].

### Data filtering and processing

The Doppler-shift method (change in frequency of the satellite signal) was used to calculate the raw GPS data [43]. The raw velocity and subsequent acceleration and metabolic power data were filtered by proprietary software (Catapult Sprint^™^ version 5.1.4) using a median filter to reduce inherent signal noise [44]. Acceleration derived via GPS used for the calculation of metabolic power was derived over a 0.2 second time interval (smoothing filter width as defined in the software). The intelligent motion filter option provided within Catapult Sprint^™^ software was not activated. The processing algorithm for PlayerLoad^™^ was unfortunately not available to authors for proprietary reasons.

Player match movement files were cropped to include only match time using Catapult Sprint^™^.

Individual player files were then exported from Catapult Sprint^™^ via comma-separated values files into Microsoft Excel 2013 (version 15, Microsoft Corp, Redmond, WA, USA) and then imported into the Statistical Analysis System (SAS, version 9.4; SAS Institute, Cary, NC) for further data processing. A program was written within SAS to identify the maximum mean value of each measure (PlayerLoad^™^, mean speed and metabolic power) using a rolling moving average of a given duration (5, 10, 20, 30, 60, 120, 300 and 600 seconds). Stringent data inclusion criteria were applied to individual player files before performing further analyses. Data inclusion criteria for individual player files included mean horizontal dilution of position (HDOP) of ≤1.5, mean number of satellites ≥4, and ≥600 seconds spent on field. Unrealistic velocity spikes ≥11 m.s^-1^ and maximum accelerations ≥6 m.s^-2^ were also removed during this process. The mean ± standard deviation (SD) number of satellites for the elite and sub-elite cohort’s data sets were 13.5 ± 1.1 and 14.3 ± 1.7 respectively, whilst HDOP was 0.9 ± 0.3 and 0.8 ± 0.2 respectively. These values are indicative of good GPS signal quality as per manufacturer’s recommendations. A total of 421 elite and 256 sub-elite player match-half files remained for further analysis.

### Statistical analyses

Each of the three measures of maximum mean movement was analysed with the general linear mixed modelling procedure (Proc Mixed) in SAS. The measures were log-transformed prior to analysis to reduce non-uniformity of error [45] and to express effects and errors in percent units after back-transformation. The fixed effects in the model were player position (backs, forwards) interacted with match-half (1^st^, 2^nd^) to provide estimates of means and differences between the means of these variables; these effects were also interacted with time on the field (numeric linear) to produce maximum mean values that adjust to the average time a player is on the field across the positions and halves. The random effects in the model were player identity (to estimate differences between player means), match identity (to estimate differences between match means), the interaction of player and match identities (to estimate changes within players between matches); with different variances estimated for the random effects for the two positions (forwards, backs). The residual in the model estimated within-athlete variability (typical error or "noise") between match halves; different residual variances were estimated for the four position*half groups and to simplify presentation were averaged. The random effects were combined into intraclass correlation coefficients (ICCs) representing reliability of each measure. Compatibility limits for the correlations were generated with a bootstrap method, in which the independent standard errors of the variances provided by the mixed model were combined with random normal deviates to generate bootstrap samples.

The magnitudes of effects (differences or changes in means; standard deviations derived from random effects) were evaluated by standardisation, which was performed by dividing each effect by the between-player standard deviation in a typical match. This standard deviation was derived for ease of calculation from four separate analysis (for each position and half) by adding the variances for the random effects for player identity and the residual, converting the resulting variances to standard deviations and deriving the harmonic mean, which provided an appropriate mean standard deviation for all pairwise comparisons of positions and halves [46]. The smallest worthwhile difference or change in means (the "signal", for comparison with "noise") is 0.2 standard deviations; thresholds for moderate, large and very large differences are 0.6, 1.2 and 2.0, respectively [45]. Thresholds for evaluating standard deviations (derived by taking square roots of random-effect variances) were half these values [47]. Typical error was evaluated via the following thresholds: < 0.5 neligable error, 0.5–1.5 small, 1.5–3 moderate, 3–6 large, 6–10 very large > 10 extremely large.

Uncertainty in effects was expressed as 90% compatibility limits and as probabilities that the true effect was substantially positive and negative (derived from standard errors, assuming a normal sampling distribution). These probabilities were used to make a qualitative probabilistic non-clinical magnitude-based decisions about the true effect [45]: if the probabilities of the effect being substantially positive and negative were both >5%, the effect was reported as unclear; the effect was otherwise clear and reported as the magnitude of the observed value, with the qualitative probability that the true effect was a substantial increase, a substantial decrease, or a trivial difference (whichever outcome had the largest probability). The scale for interpreting the probabilities was as follows: 25–75%, possible; 75–95%, likely; 95–99.5%, very likely; >99.5%, most likely.

For a sample size of approximately 50, standardised residuals (*t-*statistics) of >3.5 can be considered outliers [45]. Considering the cohort size (60 participants, 30 in each group) and the number of subsequent player files in each cohort (421 and 256 files respectively), a standardised residual outlier threshold of >3.5 was applied, with those above the threshold removed.

#### Evaluating measure sensitivity

Sensitivity of measures was quantified via evaluation of ("signal") and typical error of measurement ("noise"). The smallest worthwhile difference or change in means (the "signal", for comparison with "noise") is 0.2 standard deviations; thresholds for moderate, large and very large differences are 0.6, 1.2 and 2.0, respectively [45]. To estimate the typical error or noise of each measure, the difference between observed and predicted values (the residual) was added as a random effect in the general linear mixed model as stated previously.

#### Evaluating measure reliability

Variabilities within and between players represented reliability of each measure. The random effects of: player identity, match identity, interaction of player and match identities and the residual were combined into intraclass correlation coefficients, representing reliability of each measure. Magnitudes of ICCs were evaluated using the following thresholds: >0.99, extremely high; ≤0.99 to ≥0.90, very high; <0.90 to ≥0.75, high; <0.75 to ≥0.50, moderate; <0.50 to ≥0.20, low; <0.20, very low [48].

#### Evaluating measure construct validity

Construct validity (i.e. whether a tool measures what it is supposed to measure) of the three measures (mean speed, PlayerLoad^™^ and metabolic power) was assessed by comparing mean differences between playing positions and match halves with findings from previous rugby union time-motion analyses that use other tools (e.g. other GPS and accelerometer models, local positioning systems, optical systems, notational analysis etc.) and measures (several high-intensity metrics) to quantify a common construct (i.e. player movement). More specifically, many rugby union time-motion analyses have observed differences in high-intensity movement between playing positions and match-halves [28, 33, 36, 49–51]. If the peak intensity of competition differences observed in the present study were consistent with expected positional and match-half activity profiles from previous studies, then the measures were deemed to relate to previous findings and display construct validity. For example, if the weight of rugby time-motion analysis literature revealed that backs produce greater maximal speeds and accelerations than forwards during competition, and the mean speed and metabolic power measures used in the present study quantified similar positional differences, the measures display some level of construct validity.

## Results

Duration-specific grand means and standard deviations (SD) of each measure of maximum mean movement are shown in Tables 2, 3 & 4 to provide context for the positional differences and match-half changes.

**Table 2:** Maximum mean speed (m.min^-1^) descriptive, effect and inferential statistics for rolling epoch durations of 5 to 600-s within elite and sub-elite rugby union competition.

Epoch duration (s)	Grand mean (m.min^-1^)	Between-subject SD (%)	Typical error (%)	SWC (%)	Positional differences (backs–forwards)		Match-half change (second–first half)	
					Mean; ±90%CI (%)	Inference^a^	Mean; ±90%CI (%)	Inference^a^
*Super 15 Rugby (elite)*								
5	380	12.3	10.1	2.5	19.8; ±6.9	Large ↑****	-3.7; ±1.7	Small ↓**
10	309	13.6	12.3	2.7	18.3; ±6.7	Large ↑****	-5.4; ±2.1	Small ↓***
20	235	12.7	12.0	2.5	15.6; ±5.5	Large ↑****	-4.8; ±2.0	Small ↓***
30	201	11.0	11.1	2.2	14.5; ±5.0	Large ↑****	-5.1; ±1.9	Small ↓***
60	155	9.8	10.1	2.0	11.7; ±4.6	Large ↑****	-3.2; ±1.7	Small ↓**
120	123	9.5	9.8	1.9	8.2; ±4.9	Moderate ↑***	-2.6; ±1.7	Small ↓**
300	91	10.6	11.1	2.1	7.8; ±5.4	Moderate ↑***	-6.6; ±1.8	Moderate ↓****
600	76	9.7	9.9	1.9	9.1; ±6.3	Moderate ↑***	-5.7; ±1.7	Moderate ↓****
*National Rugby Championship (sub-elite)*								
5	387	13.6	11.5	2.7	22.3; ±9.6	Large ↑****	-3.8; ±5.8	Small ↓*
10	320	14.1	12.1	2.8	22.9; ±10.5	Large ↑****	-1.6; ±6.3	Trivial
20	236	12.3	11.3	2.5	22.3; ±9.4	Large ↑****	2.1; ±5.9	Small ↑
30	201	11.8	10.0	2.4	13.7; ±8.8	Moderate ↑***	0.6; ±5.4	Trivial
60	158	10.0	8.9	2.0	10.2; ±7.3	Moderate ↑***	-3.6; ±3.7	Small ↓**
120	128	10.3	9.4	2.1	8.9; ±7.6	Moderate ↑**	-6.2; ±4.1	Moderate ↓***
300	98	10.0	9.5	2.0	4.3; ±8.4	Small ↑	-2.3; ±4.8	Small ↓
600	82	10.1	8.0	2.0	2.7; ±7.6	Small ↑	-6.2; ±3.7	Moderate ↓***

Grand means represent the mean of pooled positional (backs, forwards) and match-half (first, second) data.

SWC, smallest worthwhile change (0.2 of between-subject SD); 90%CI, 90% compatibility interval.

^a^Inferences specify the magnitude, direction and likelihood of the true value of clear effects. Magnitudes were defined by standardisation (see text). Likelihood for clear trivial effects: ^0^possible, ^00^likely, ^000^very likely. Likelihood for clear substantial effects: *possible, **likely, ***very likely, ****most likely.

**Table 3:** Maximum mean metabolic power (W.kg^-1^) descriptive, effect and inferential statistics for rolling epoch durations of 5 to 600-s within elite and sub-elite rugby union competition.

Epoch duration (s)	Grand mean (W.kg^-1^)	Between-subject SD (%)	Typical error (%)	SWC (%)	Positional differences (backs–forwards)		Match-half change (second–first half)	
					Mean; ±90%CI (%)	Inference^a^	Mean; ±90%CI (%)	Inference^a^
*Super 15 Rugby (elite)*								
5	54.9	16.8	13.1	3.4	30.0; ±10.1	Large ↑****	-1.5; ±2.3	Trivial ^00^
10	41.2	16.7	13.7	3.3	29.3: ±9.4	Large ↑****	-4.3; ±2.3	Small ↓**
20	29.9	15.1	13.1	3.0	24.6; ±7.3	Large ↑****	-4.3; ±2.2	Small ↓**
30	25.0	12.7	11.9	2.5	23.5; ±6.1	Large ↑****	-4.6; ±2.0	Small ↓***
60	18.7	12.1	11.0	2.4	17.3; ±6.4	Large ↑****	-3.6; ±1.9	Small ↓**
120	14.3	11.8	10.6	2.4	11.8; 6.6	Moderate ↑***	-3.3; ±1.8	Small ↓**
300	10.3	12.5	12.0	2.5	11.6; ±6.5	Moderate ↑***	-7.0; ±2.0	Moderate ↓****
600	8.4	11.7	11.6	2.3	11.9; ±7.0	Moderate ↑***	-7.0; ±2.0	Moderate ↓****
*National Rugby Championship (sub-elite)*								
5	54.2	16.6	13.6	3.3	34.8; ±13.6	Large ↑****	-6.8; ±6.3	Small ↓**
10	41.4	17.2	14.2	3.4	35.9: ±14.3	Large ↑****	-4.9; ±6.8	Small ↓*
20	29.0	14.3	12.2	2.9	35.6: ±12.2	V. Large ↑****	1.1; ±6.6	Trivial
30	23.7	13.0	9.6	2.6	28.5: ±11.1	V. Large ↑****	3.9; ±5.6	Small ↑*
60	18.3	11.5	9.5	2.3	19.4; ±9.0	Large ↑****	-4.3; ±4.3	Small ↓**
120	14.4	11.3	10.4	2.3	17.0; ±8.9	Large ↑****	-7.7; ±4.7	Moderate ↓***
300	10.9	10.6	9.9	2.1	8.8; ±9.4	Moderate ↑**	-4.6; ±4.9	Small ↓**
600	9.0	11.2	8.7	2.2	4.9; ±8.9	Small ↑	-7.3; ±4.0	Moderate ↓***

Grand means represent the mean of pooled positional (backs, forwards) and match-half (first, second) data.

SWC, smallest worthwhile change (0.2 of between-subject SD); 90%CI, 90% compatibility interval.

^a^Inferences specify the magnitude, direction and likelihood of the true value of clear effects. Magnitudes were defined by standardisation (see text). Likelihood for clear trivial effects: ^0^possible, ^00^likely, ^000^very likely. Likelihood for clear substantial effects: *possible, **likely, ***very likely, ****most likely.

**Table 4:** Maximum mean PlayerLoad^™^ descriptive, effect and inferential statistics for rolling epoch durations of 5 to 600-s within elite and sub-elite rugby union competition.

Epoch duration (s)	Grand mean (au)	Between-subject SD (%)	Typical error (%)	SWC (%)	Positional differences (backs–forwards)		Match-half change (second–first half)	
					Mean; ±90%CI (%)	Inference^a^	Mean; ±90%CI (%)	Inference^a^
*Super 15 Rugby (elite)*								
5	3.8	15.9	13.5	3.2	7.4; ±6.5	Small ↑**	-0.8; ±2.4	Trivial ^00^
10	2.8	15.3	12.1	3.1	7.0; ±6.8	Small ↑**	-1.2; ±2.2	Trivial ^00^
20	2.0	13.6	11.0	2.7	1.9: ±5.8	Small ↑	-0.2; ±0.2	Trivial ^000^
30	1.8	12.2	9.8	2.4	-0.9; ±5.2	Trivial	-0.2; ±1.8	Trivial ^000^
60	1.4	12.7	8.7	2.5	-5.3; ±5.9	Small ↓**	-0.2; ±1.7	Trivial ^000^
120	1.1	13.6	9.8	2.7	-11.3; ±6.3	Moderate ↓***	0.1; ±1.8	Trivial ^000^
300	0.7	13.9	10.2	2.8	-11.4; ±7.2	Moderate ↓***	-2.5; ±1.8	Small ↓*
600	0.6	14.1	9.8	2.8	-11.9; ±7.7	Moderate ↓***	-1.8; ±1.7	Trivial ^00^
*National Rugby Championship (sub-elite)*								
5	3.5	15.5	14.0	3.1	15.5; ±10.1	Moderate ↑***	2.5; ±6.8	Small ↑
10	2.7	15.6	14.4	3.1	12.6; ±9.7	Moderate ↑***	-2.7; ±6.6	Small ↓
20	2.0	12.9	12.1	2.6	10.1; ±8.2	Moderate ↑**	-0.9; ±6.2	Trivial
30	1.6	10.6	9.7	2.1	8.5; ±6.9	Moderate ↑**	2.8; ±5.1	Small ↑
60	1.3	9.3	8.5	1.9	0.6; ±5.0	Trivial	-8.3; ±3.5	Moderate ↓****
120	1.0	10.0	10.5	2.0	-3.7; ±6.3	Small ↓	-12.4; ±4.3	Large ↓****
300	0.7	9.5	9.7	1.9	-6.9; ±6.9	Moderate ↓**	-8.8; ±4.4	Moderate ↓***
600	0.6	10.0	8.1	2.0	-10.4; ±6.8	Moderate ↓***	-11.8; ±3.7	Large ↓****

Grand means represent the mean of pooled positional (backs, forwards) and match-half (first, second) data.

SWC, smallest worthwhile change (0.2 of between-subject SD); 90%CI, 90% compatibility interval.

^a^Inferences specify the magnitude, direction and likelihood of the true value of clear effects. Magnitudes were defined by standardisation (see text). Likelihood for clear trivial effects: ^0^possible, ^00^likely, ^000^very likely. Likelihood for clear substantial effects: *possible, **likely, ***very likely, ****most likely.

### Sensitivity of measures

Global positioning system and accelerometer measures had poor sensitivity for quantifying maximum mean movement across all epochs and both levels of competition, with noise 4× to 5× the signal (Tables 2, 3 & 4).

### Within-match, between-half reliability

Maximum mean movement measured via GPS- and accelerometer-derived measures displayed very low to low within-match, between-half reliability (ICC range; 0.0 to 0.5) during both sub-elite and elite rugby union match-play. Maximum mean PlayerLoad^™^ displayed higher within-match, between-half reliability in the elite cohort than either mean speed or metabolic power for epoch durations ≥60-s, although this test-retest reliability was still low (ICC; ~0.4). For the sub-elite backs, mean speed and metabolic power generally had slightly higher ICCs (~0.4) than PlayerLoad^™^ (~0.2) for epochs ≥30-s.

### Between-match, within-half reliability

Reliability of maximum mean movement within a specific match-half from match-to-match was generally very low to low for all measures (ICC <0.5), with movement reliability typically increasing with rolling epoch duration. PlayerLoad^™^ generally had lower reliability when compared to either GPS-derived measure for both sub-elite forwards and backs for epoch durations ≥60-s in the 2^nd^ match-half. However, during elite match-play maximum mean movement quantified by accelerometer-derived PlayerLoad^™^ generally had higher between-match, within-half reliability when compared to mean speed and metabolic power. As the epoch duration increased in the elite cohort, so too did the reliability of maximum mean PlayerLoad^™^, with moderate to high ICCs for both positions and match halves for the 300 and 600-s epochs respectively (ICC range; 0.5 to 0.8).

### Playing position differences in maximum mean movement

**Fig 1:**
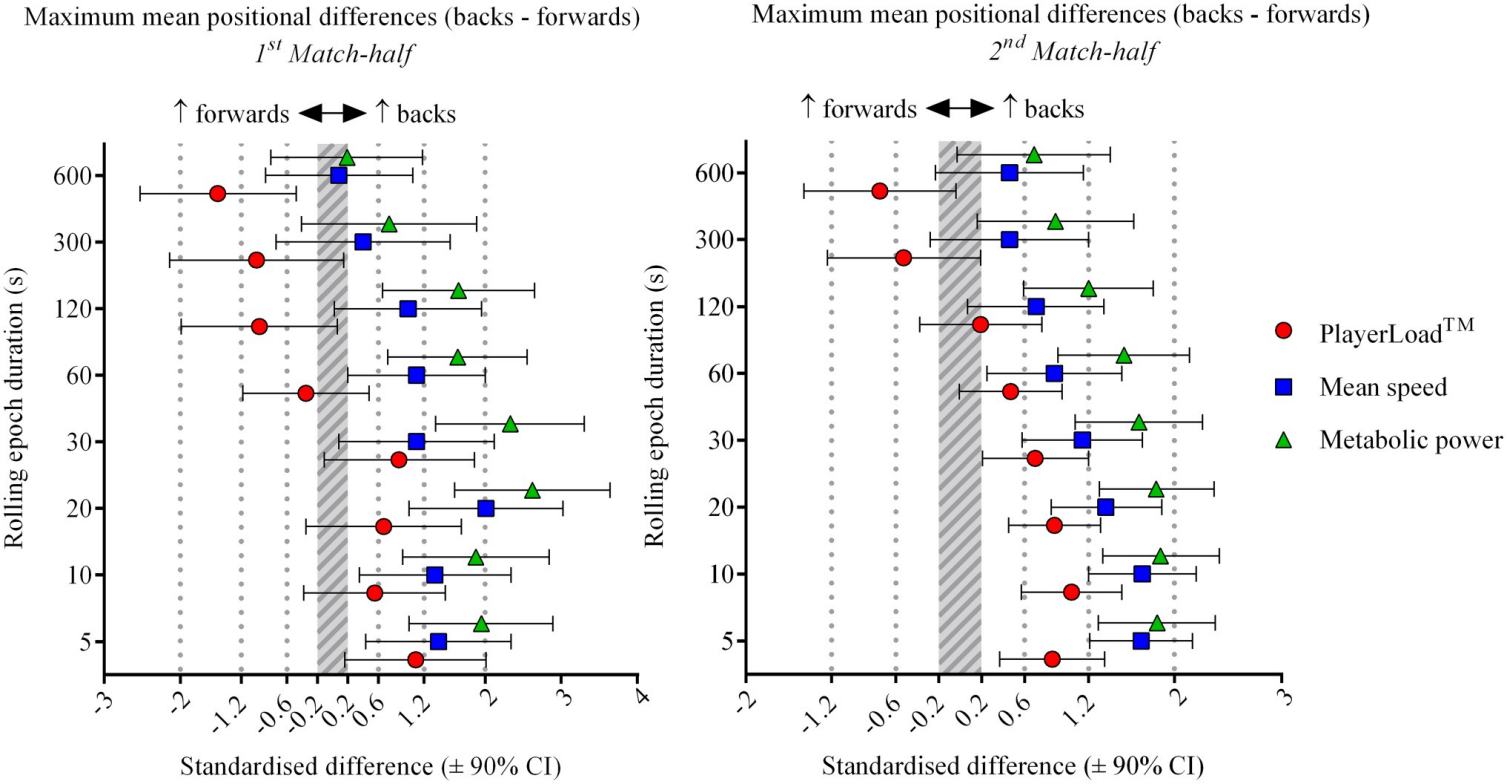
Sub-elite (National Rugby Championship) maximum mean standardised positional differences (backs–forwards) for rolling epoch durations of 5 to 600-s by match-half (1^st^ and 2^nd^ match halves).

**Fig 2:**
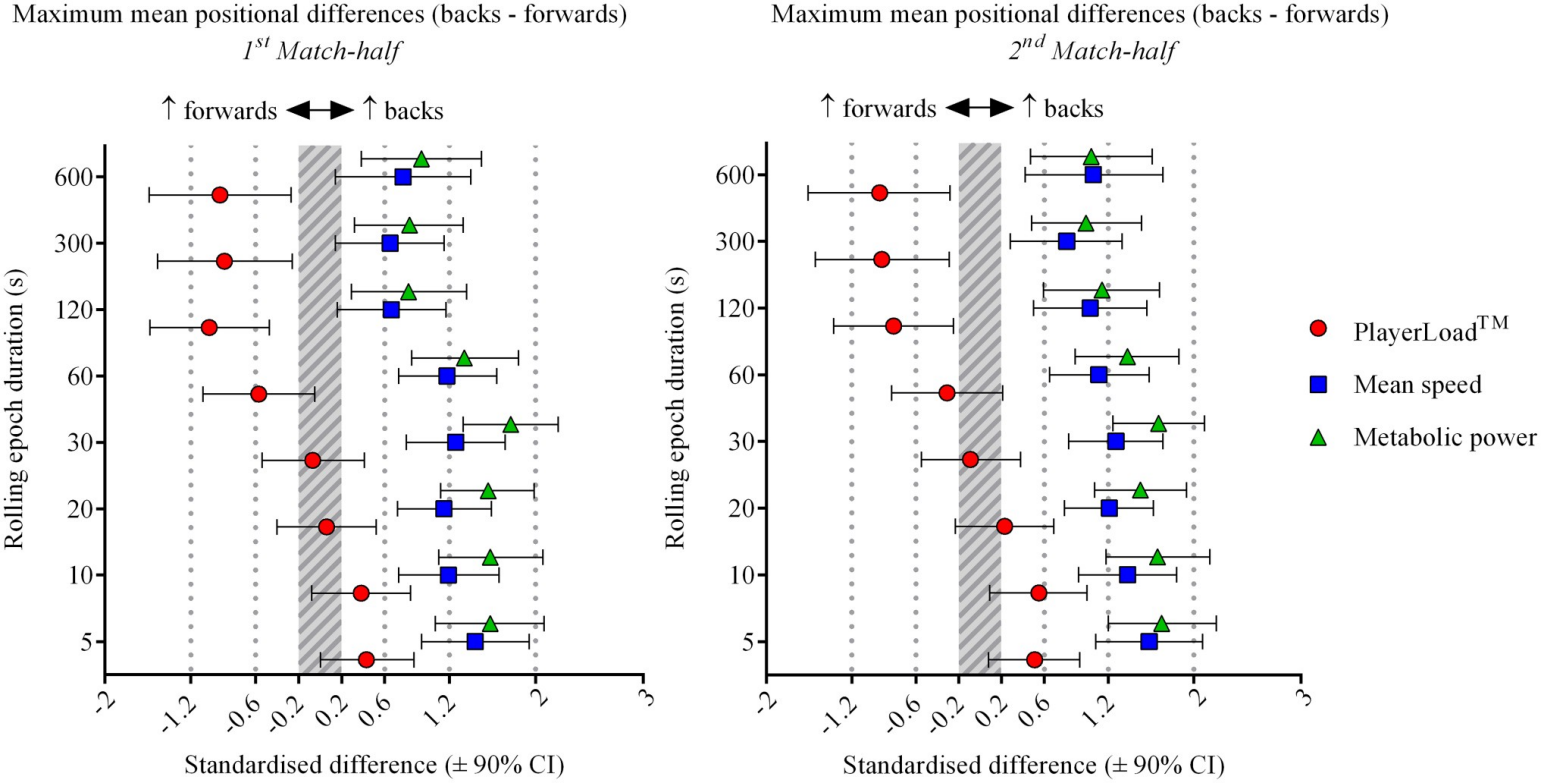
Elite (Super 15 rugby) maximum mean standardised positional differences (backs–forwards) for rolling epoch durations of 5 to 600-s by match-half (1^st^ and 2^nd^ match halves).

Relative to the backs, forwards had greater accelerometer-derived PlayerLoad^™^ per unit of distance covered or metabolic power across all rolling epochs (5 to 600-s), during both elite and sub-elite rugby union match-play (Figs 1 & 2). Elite backs produced clearly greater maximum mean speed and metabolic power compared to elite forwards for all rolling epoch durations (Table 2). A similar result was observed for the sub-elite cohort, with backs producing moderate to large higher mean speeds and large to very large higher metabolic power compared to the forwards for the shorter duration epochs of 5 to 30-s (Tables 2 & 3). However, there were *unclear* positional differences as quantified by GPS-derived measures for the longer duration 300 and 600-s epochs (Fig 1). Metabolic power consistently estimated higher maximum mean standardised differences between positions when compared to mean speed for both levels of competition and match halves, whilst also estimating larger opposing positional differences to PlayerLoad^™^ when compared to mean speed (Figs 1 & 2). As the rolling epoch duration increased from 5 to 600-s, positional differences between backs and forwards decreased (especially for the sub-elite cohort), with an evident divergence of maximum mean movement as measured by accelerometer-derived PlayerLoad^™^ when compared to GPS-derived mean speed and metabolic power post the 30-s epoch for both levels of competition (Figs 1 & 2). Elite forwards produced *very likely* greater maximum mean PlayerLoad^™^ compared to the backs (moderate effects) for longer epoch durations of 120 to 600-s across both match halves (Fig 2 & Table 4). Conversely, for the same epoch durations (120 to 600-s) the maximum means for GPS-derived measures of mean speed and metabolic power were *likely* to *most likely* higher for the backs compared to the forwards (Tables 2 & 3). Sub-elite forwards produced *likely* to *very likely* greater PlayerLoad^™^ compared to the backs for the 300 and 600-s epochs (small to large effects), compared to mostly *unclear* positional differences as quantified by GPS-derived measures (Fig 1 & Table 4).

### Match-half changes in maximum mean movement

**Fig 3:**
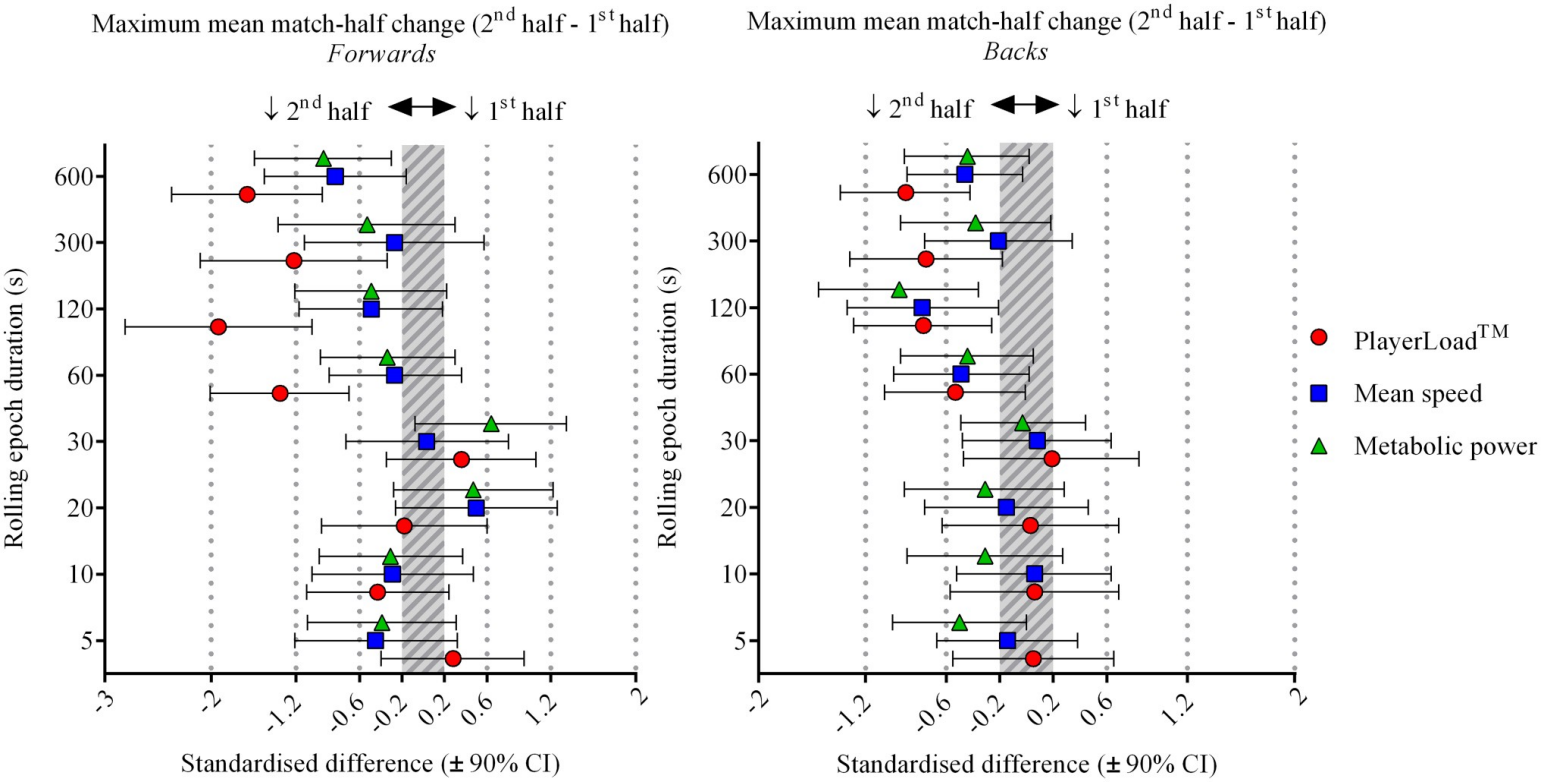
Sub-elite (National Rugby Championship) maximum mean match-half standardised changes (2^nd^ match-half - 1^st^ match-half) for rolling epoch durations of 5 to 600-s by position (forwards and backs).

**Fig 4:**
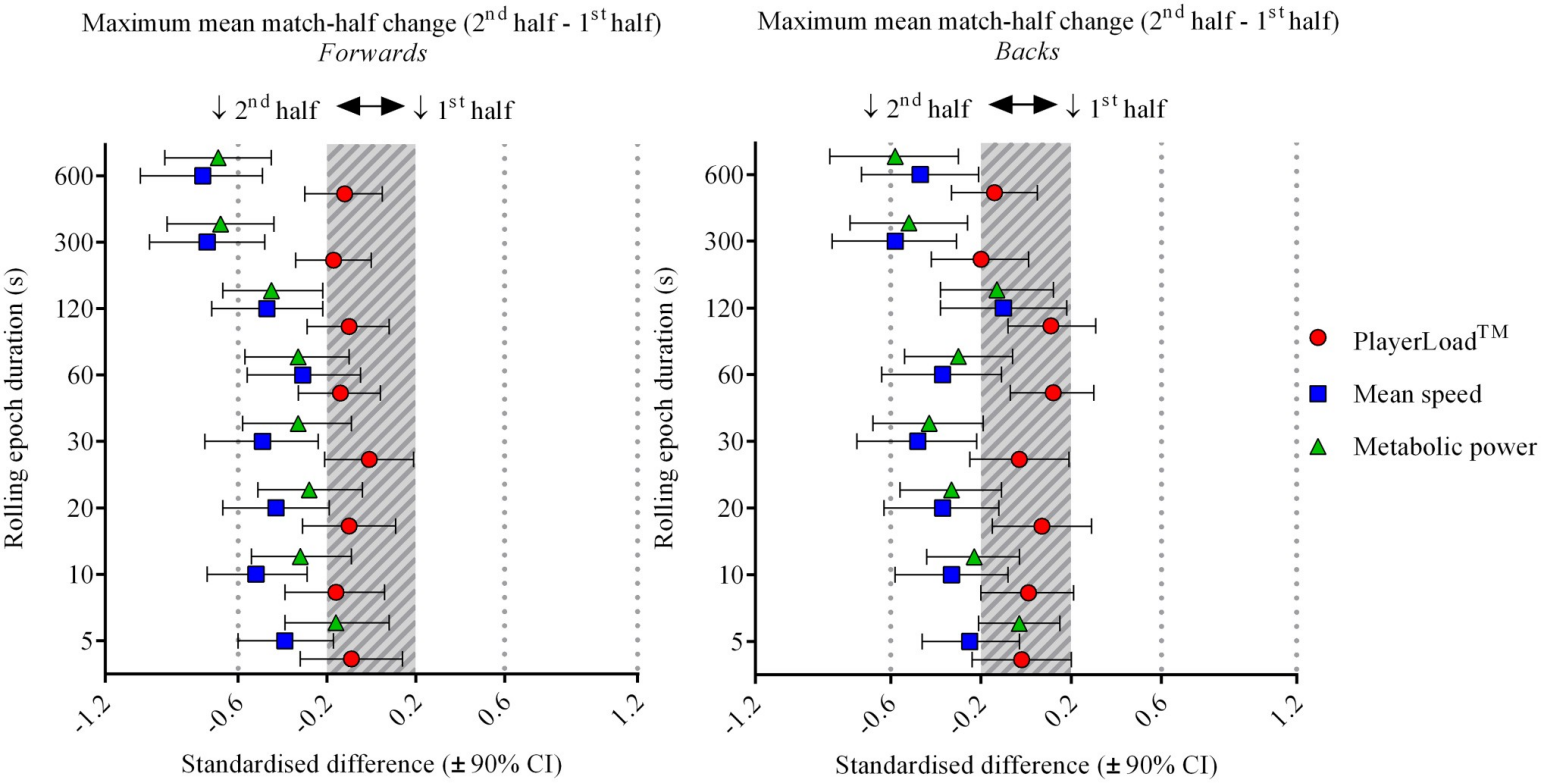
Elite (Super 15 rugby) maximum mean match-half standardised changes (2^nd^ match-half—1^st^ match-half) for rolling epoch durations of 5 to 600-s by position (forwards and backs).

Sub-elite match-half declines in maximum mean movement were more adequately quantified with accelerometer-derived PlayerLoad^™^ when compared to either GPS measure, with clearer and larger effects for epoch durations ≥60-s (Fig 3). For example, sub-elite forwards had large reductions in PlayerLoad^™^ during the 2^nd^ match-half for epoch durations ≥60-s, whilst mean speed and metabolic power half changes were mostly *unclear* across comparable durations (Fig 3, panel A and Table 4). Sub-elite match-half changes were mostly *unclear* for all measures and both positions for epoch durations of 5 to 30-s (Fig 3). Conversely during elite match-play, GPS-derived measures quantified larger and clearer 2^nd^ match-half declines in maximum mean movement than accelerometer-derived PlayerLoad^™^ (Fig 4). PlayerLoad^™^ displayed trivial or *unclear* match-half changes for both positions and across all epoch durations during elite match-play (Fig 4). Maximum mean mean speed and metabolic power generally declined in the 2^nd^ match-half by a small to moderate standardised extent, with the longer duration epochs of 300 and 600-s displaying the largest 2^nd^ match-half declines (Fig 4). Reductions in 2^nd^ match-half maximum mean movement were more evident for forwards than for backs for longer duration epochs during both sub-elite (≥60-s) and elite (≥300-s) match-play (Figs 3 & 4).

## Discussion

Several key and novel findings were observed: (1) Both GPS- and accelerometer-derived measures had poor sensitivity for quantifying rugby union maximum mean movement of 5 to 600-s; (2) To obtain adequate precision for assessing individual differences or changes in maximum mean rugby union match movement, practitioners require ~16 full matches of player movement data (approximate length of a team-sport season); (3) Maximum mean movement of 5 to 600-s was inherently unreliable, with typically low to very low within-match, between-half reliability and between-match, within-half reliability during both elite and sub-elite match-play across all measures; (4) All measures displayed construct validity by quantifying similar movement differences between playing positions and match halves to previous rugby union time-motion analyses; (5) Relative to the backs, forwards had greater PlayerLoad^™^ per unit of distance covered or metabolic power across all rolling epochs during both elite and sub-elite rugby union match-play; (6) Backs produced clearly greater maximum mean speed and metabolic power compared to forwards for all epoch durations for elites, and all epochs except for 300 and 600-s for sub-elites; (7) Elite and sub-elite forwards produced clearly greater maximum mean PlayerLoad^™^ than backs during longer (≥300-s) epoch durations; (8) Larger 2^nd^ match-half declines in maximum mean movement were evident as epoch duration increased, with greater declines for forwards than backs; (9) Maximum mean PlayerLoad^™^, mean speed and metabolic power of 5 to 600-s was of substantially higher intensity than previously reported rugby union whole-period match averages; and (10) Rolling epoch analysis of less than 1-minute (i.e., 5, 10, 20, 30-s) provided useful data that may inform high-intensity interval training prescription and monitoring. The remainder of the discussion focusses on interpretation and application of our findings in the context of others.

### Sensitivity of measures

Both GPS- and accelerometer-derived measures had poor sensitivity for quantifying athlete movement during the most intense passages of rugby union match-play. A professional rugby union athlete’s maximum mean movement of 5 to 600-s during match-play will vary on average from measurement to measurement by ~11% (mean range; 8 to 14%). This typical error of measurement or noise represents the error that practitioners must contend with when assessing individual differences or changes in athlete movement. The noise (~11%) can then be compared to smallest worthwhile change or signal (mean SWC of 2.5%) to calculate the number of repeated measurements required to attain adequate precision of estimates. If signal equal to the noise was deemed acceptable precision by a practitioner, then using our data as an example, approximately 16 measurements (16 match halves or 8 total matches) would be required to reduce the noise 4 fold to ~2.5%. However, for more adequate precision of estimates ideally the noise should be half that of the signal [48]. Thus practitioners of professional rugby union athletes require ~32 match-half measurements or ~16 full matches of player movement data (approximate length of a team-sport season) to obtain adequate precision (i.e., noise half that of the signal) for assessing individual differences or changes in maximum mean match movement of 5 to 600-s. It is our hope that in the future more researcher’s will report the sensitivity of investigated external load measures to inform practitioners on the number of measurements required to accurately interpret and confidently act on movement data.

### Reliability of measures

Rugby union maximum mean movement of 5 to 600-s was inherently unreliable, with typically low to very low within-match, between-half reliability and between-match, within-half reliability during both elite and sub-elite match-play across all measures (ICC; <0.50). Similarly, in English Championship professional rugby union matches, within- and between-player variability of high-intensity activity was large whilst player “match load” variables such as PlayerLoad^™^ provided a more stable measure of between-match player movement with a coefficient of variation of ~10% [52]. In agreeance, we found that maximum mean PlayerLoad^™^ displayed improved between-match, within-half reliability (moderate to high) when compared to either mean speed or metabolic power (low) as epoch duration increased past 60-s during elite rugby union match-play (up to ICC; 0.8). PlayerLoad^™^ may therefore be a more reliable and stable measure of external load than mean speed or metabolic power for monitoring elite rugby union athletes during longer training drills or match bouts (e.g., match-half or whole match).

Our data provides further evidence that reliability of team-sport movement as measured by both GPS and accelerometers is inversely related to speed of movement. This finding creates a dilemma for practitioners when selecting measures and is in accordance with the suggestion that validity and reliability of a measure is likely inversely related to its importance for external load quantification and monitoring [40, 53]. Low measure reliability does not mean that PlayerLoad^™^, mean speed and metabolic power should not be used in the context of quantifying and monitoring maximum mean movement, but rather suggests that more caution is needed when interpreting individual differences or changes. Defining a larger and more conservative SWC [54] and/or having more repeated measures are possible solutions to this dilemma, as highlighted by our poor measure sensitivity and correspondingly low reliability findings.

### Playing position differences in maximum mean movement

As expected, GPS was unable to quantify all forms of external load experienced by elite and sub-elite rugby union athletes during the most intense periods of match-play, particularly underestimating external load of the forwards. Forwards are primarily responsible for engaging in contests for possession involving contact such as scrums, lineouts, rucks, mauls and tackles, whilst backs are primarily tasked with trying to gain territory and score points [55]. Compared to rugby union backs, forwards have increased frequency of impacts [56], “static” exercise bouts (i.e., scrums, rucks & mauls) and durations of “static” bouts [57]. During competition forwards also produce greater mean acceleration than backs [58] and “aggregated accelerometer body demands” [59]. Our accelerometer-derived PlayerLoad^™^ findings corroborate with rugby union time-motion analysis positional movement differences, demonstrating the construct validity of accelerometers to quantify many frequently occurring rugby union movements that are underestimated by GPS. If the present rolling epoch analysis findings of 5 to 600-s were to be extrapolated in duration to well beyond 600-s (e.g., 40-min match-half) that typically occurs in practice, then the gradual accumulation of many sport-specific and collision-based movements that incur little horizontal displacement over time would likely result in further underestimation of external load via the sole use of GPS. We recommend that practitioners use accelerometers alongside GPS to more adequately quantify, monitor and prescribe totality of athlete movement (external load) during collision-based team sports such as rugby union.

Over shorter exercise bout durations, backs produced greater intensity of movement than forwards. Elite backs produced greater mean speed and metabolic power compared to forwards for all durations of 5 to 600-s (ES >0.60), with similar results for the sub-elite cohort with exception of the 300 and 600-s yielding unclear positional differences. Consistent with our findings, outside backs and half-backs cover greater peak relative running distances than tight 5 forwards (front row and locks, ES >0.60) for rolling average durations of 1 to 10-mins [36]. Facilitating backs ability to produce higher peak running intensities during match-play is greater recovery time to regenerate energy stores between efforts compared to forwards, with a mean exercise: rest ratio of 1: 8.5 vs 1: 6.5 respectively. Increased rest time between efforts is largely due to forwards spending ~33% of their time exercising throughout a match completing “static” movements (e.g., scrums, rucks and mauls) compared to ~8% for backs [58]. Relative to forwards, backs complete a greater frequency of accelerations and decelarations [59], contributing to *likely* greater metabolic power for outside backs and half-backs when compared to the tight 5 (ES range; 0.86 to 0.99) [36]. These findings are not surprising considering backs are required to evade opponents with rapid acceleration, change of direction and/or maximal speed to score tries or chase down and tackle opponents to deny try scoring, but do highlight the need for position specific training prescription and monitoring. Practitioners may alter the playing area, number of players, rules and the duration of small-sided games to modify the frequency and intensity of player movements to achieve desired position-specific activity profiles. For example, larger small-sided game playing areas with less players will facilitate more high-speed running whilst smaller playing areas with more players will facilitate more acceleratory, change of direction and collision-based movements.

When compared to our Super 15 Rugby cohort findings, international rugby union players cover more distance yet accelerate less during the most intense periods of competition [36]. International test-match players thus may complete a greater amount of “constant” speed running compared to Super 15 players, who run at a lower mean speed, yet accelerate more during maximum mean periods of activity as reflected by the increased metabolic power production. Super 15 mean speeds may be comparably lower than test-match rugby because our analysis provided a maximum value for each match-half, whilst the test-match analysis produced one maximum value from the entire match [36]. Maximum mean movement differences between investigations may also be attributed to level of competition, team and opposition playing styles, technologies used (Catapult Optimeye S5 vs GPSports SPI HPU units) and statistical analyses performed (e.g., fixed and random effects within model, data inclusion/exclusion criteria etc.). Movement comparisions between levels of competition may help performance staff to prescribe competition level match-specific training intensities to their athletes who are “handed-over” from professional club to national team environments and vice-versa.

Our findings provide some evidence to support the construct validity of mean speed and metabolic power for quantifying positional differences during the most intense periods of elite and sub-elite rugby union match-play. Mean speed and metabolic power positional differences of this study are in agreeance with prior rugby union investigations [36], positional playing roles, and the metabolic power theoretical model accounting for both velocity and acceleration based events. Our findings help to improve limited understanding of position- and duration-specific peak energy expenditures of professional rugby union competition. We do however recognise the many limitations of the metabolic power method for estimating energy cost during intermittent natured team-sport movement. Metabolic power grossly underestimates movement during shuttle running by 13 to 16% [60], a soccer-specific circuit by 29% [14], a generalised team-sport circuit by ~44% [61] and a rugby-specific circuit by ~45% [42], underpinning its lack of criterion validity versus portable gas analysers. Given metabolic power’s sensitivity and reliability to quantify movement differences was no better than the other investigated measures, metabolic power data (W.kg^-1^) are hard to prescribe team-sport training from, and many poor criterion validity findings from previous literature, we advise caution with its use.

### Match-half changes in maximum mean movement

Our findings suggest that professional rugby union athletes preserve their ability to complete maximal intensity movement over shorter durations (≤30-s) across match halves by reducing the amount of movement they perform at lower relative intensities. Similar declines in lower relative intensity cruising and striding distances have been reported as match-half duration progresses and between match halves during rugby union competition [49]. Reduced running “performance” across the course of a match has been proposed to broadly identify physiological impairment of a player, suggestive of acute fatigue [62]. Gradual declines in running intensity throughout match-play are suggestive of players adopting a “slow-positive” pacing profile, common amongst many team-sport activity profiles [63]. Whilst longer duration efforts of lower relative intensity generally declined in the 2^nd^ match-half (up to 12%), efforts of shorter duration and higher relative intensity (≤30-s) exhibited trivial or small match-half reductions. The lack of decline in very high-intensity rugby union movement between halves is similar to other elite rugby union time-motion analysis findings reporting “no change” in work-to-rest ratios [58] and high-intensity running [57] between halves. Equally, high-intensity movements (high-intensity running, sprinting, maximal accelerations, repeated high-intensity efforts and contacts) did not substantially decrease between halves during professional rugby union as quantified by GPS and integrated inertial sensors [49]. Consensus on match-half changes in very high-intensity movement between our findings and other investigations across both GPS and accelerometer measures and within both elite and sub-elite rugby union competition demonstrates that the investigated wearable technology measures display construct validity in measuring what they “ought” to measure. Duration- and position-specific match-half change data may improve our understanding of athlete pacing strategies and this information may then be used to inform substitution/rotation decisions.

### Relationship between accelerometer and GPS measures

It was clear from the pattern of positional differences across epoch durations and levels of competition that GPS and accelerometer measures provided different information about rugby union player movement. These findings demonstrate that use of either GPS or accelerometers in isolation is inadequate to accurately quantify all forms of rugby union external load. Our findings support a recent training load monitoring framework for team sports that separates physiological and biomechanical load-adaptation pathways [64]. This framework uses an analogy of a car to describe the physiological vs biomechanical external load that team-sport athletes experience. The physiological load component can be viewed as a car engine with GPS time, distance and speed derivatives providing an estimate of “fuel” in the player’s “engine”, facilitating monitoring of external work to estimate internal energy demands or metabolic load (e.g., glycogen depletion, heart rate). Whereas biomechanical load refers to external work performed by the body’s soft tissues (e.g., muscles, bones and ligaments, analogous to a car’s suspension) against the ground and other player’s during impact, that can be estimated in the field with highly responsive motion sensors such as accelerometers.

PlayerLoad^™^ has strong positive correlations with total distance in Australian Rules Football [*r* = 0.63 to 0.76; [65] and *r* = 0.90; [26]], and is mainly derived from vertical axis accelerations (44.1% ± 2.5%) during Australian Rules Football match-play [25] and treadmill running (55.7% ± 5.3%) [66]. These results make intuitive sense as team-sport athletes run great distances leading to a high frequency of propulsive and breaking forces against the ground that are quantified by accelerometers as vertical accelerations accumulated over time. Variations in physiological and biomechanical loads are generally experienced together [64], hence why total distance and PlayerLoad^™^ often correlate. Our results indicate that neither accelerometer nor GPS measures should be used a proxy measure for the other when attempting to quantify the most intense periods of collision-based team-sport match-play as whether they are correlated or not, they clearly measure different constructs. Furthermore, the common use of total distance as a proxy measure of overall training and/or match volume [53] should be undertaken with caution and is not advised when monitoring and prescribing rugby union athlete external training loads. If practitioners want to understand both the physiological and biomechanical external loads of their athletes for informing subsequent recovery and training design, both GPS and accelerometers should be used.

No research using optical or local positioning systems to quantify player movement has utilised the methodological approach outlined in the present study, although this could be easily achieved in future investigations. All that would be required are player positional x and y coordinates for these alternate player tracking solutions to calculate distance, velocity, acceleration and subsequently replicate mean speed and metabolic power measures we used. Global positioning systems may calculate velocity via positional differentiation (change in device location with each satellite signal) or using the Doppler-shift method (change in frequency in the satellite signal). Most GPS manufacturers now use the Doppler-shift method as it has been reported to have greater precision and reduced measurement error [43] when compared to deriving velocity via distance over time calculations that optical and local positioning systems use. Another key advantage of wearable systems is the 3-dimensional (x, y and z) quantification of athletic movement via integrated accelerometry, highlighted by our findings suggesting that athlete external loads will be underestimated if only movement in x and y coordinates are measured, making intuitive sense.

### Implications for training prescription

The most intense periods of rugby union match-play were of substantially higher intensity than previously reported whole-period match averages. For example, Super 15 rugby union forwards and backs produced match mean speeds of 56.1 and 68.7 m.min^-1^ respectively [67]. These whole match mean speed numbers are lower than the longest epoch duration (600-s) maximum means of elite Super 15 forwards (72.3 m.min^-1^) and backs (79.1 m.min^-1^) (Table 2). Grand mean speed reached 155 m.min^-1^ during the maximum mean 60-s epoch and 380 m.min^-1^ during the most intense 5-s epoch (Table 2). Similar stark discrepancies between movement intensities can be observed when comparing the 5 to 600-s maximum means of PlayerLoad^™^ and metabolic power herein compared to previous rugby union investigations quantifying whole-period averages. Our data illustrates that if professional rugby union training is prescribed relative to the average activity profile of a match, players will be under-prepared for the most intense periods or “worst case scenarios” of match-play.

Not surpringly our data indicate that as exercise duration increases, intensity of rugby union match movement declines. The declines in movement are non-linear and logarithmic in nature, with this intensity—duration physiological relationship often referred to as the power-law relationship [38]. The power-law relationship will be further explored in an upcoming manuscript of ours, however for the purpose of the present investigation it was clear that although there are many complex interactions between central and peripheral fatigue and numerous contextual match factors inherent within team sports, movement duration is still very predictive of movement intensity. Subsequently, practitioners may use mathematical modelling of the power-law relationship to predict movement intensity over a range of durations outside of those collected by wearable technology with reasonable accuracy, enabling practitioners to prescribe training that is more specific to the physiological and biomechanical rigors of competition.

### Strengths, limitations & future directions

Whilst this investigation provides many novel and meaningful insights that may aid coaching and performance staff in quantifying, monitoring and prescribing athlete external loads, there are limitations that need to be acknowledged. Positional analyses were limited to positional forward and back packs rather than more specific playing positions (e.g., prop, centre, scrum-half) to increase precision of estimates and to first assess if the respective technologies were sensitive enough to quantify broader positional classifications prior to comparing specific positional groupings. The case study nature of the present study may be considered a limitation and whilst two professional teams of two competitive levels with many repeated measures were included, league-wide investigations with opposition analyses is the way forward to better understand collision-based team-sport activity profiles. We acknowledge that placement of an accelerometer to the trunk is only an estimate of whole-body accelerations that is far from perfect, although offers a starting point for biomechanical load estimation in the field. Future research should continue to investigate the influence of sensor location, sensor harnessing and relationships between segmental and whole-body acceleration. Future research should integrate and overlay video analysis with activity profile wearable technology data to better understand how many individual and match contextual factors influence duration-specific maximum mean movement of individuals and the team as a whole. Further, whether these intense periods of match-play have any bearing on individual and/or team key performance indicators and/or match outcome is unclear. Understanding of player movement immediately following these maximum mean periods of activity is also limited and may provide valuable insights on player pacing strategies and accumulated or transient fatigue that may inform real-time player substitution or rotation decisions. Altogether, knowledge of the strengths and limitations of the technologies and the measures they provide is crucial for both practitioners and researchers alike to accurately interpret the external load data produced and subsequently provide recommendations for action to influence the training process.

### Practical applications

- Professional rugby union player movement needs to be monitored across many matches to obtain adequate precision for assessing individuals during intense periods of match-play.
- Accelerometers should be used in addition to GPS to quantify, monitor and prescribe player movement in rugby union and other collision-based team sports.
- Neither accelerometer nor GPS measures should be used a proxy measure for the other, as they measure different external load constructs (biomechanical and physiological load respectively).
- Duration- and position-specific player movement data derived from wearable technologies and rolling epoch analyses may be used as a reference for training monitoring and prescription to objectively prepare players for the most intense periods of competition. For example, small-sided games may be modified (pitch size, number of players, rules, verbal encouragement) to achieve desired duration- and position-specific physiological and biomechanical external loads whilst simultaneously training technical and tactical skills.
- Given metabolic power’s sensitivity and reliability to quantify movement differences was no better than the other investigated measures, and metabolic power data are hard to prescribe team-sport training from, we advise caution with its use.

## Conclusions

The poor sensitivity and low reliability of GPS and accelerometer measures of maximum mean movement imply that rugby union players need to be monitored across many matches to obtain adequate precision for assessing individuals. Although all measures displayed construct validity, accelerometers provided meaningful information additional to that of GPS. We recommend that practitioners use accelerometers alongside GPS to quantify, monitor and prescribe player movement in rugby union and other collision-based team sports.

## Supporting information

S1 Data (XLSX)

## References

[1] Lachow I. The GPS dilemma: balancing military risks and economic benefits. International Security. 1995;20(1):126–48.

[2] Gallo T, Cormack S, Gabbett T, Williams M, Lorenzen C. Characteristics impacting on session rating of perceived exertion training load in Australian footballers. Journal of sports sciences. 2015;33(5):467–75. 10.1080/02640414.2014.947311 . 25113820

[3] Delaney JA, Scott TJ, Thornton HR, Bennett KJ, Gay D, Duthie GM, et al Establishing duration-specific running intensities from match-play analysis in rugby league. International journal of sports physiology and performance. 2015;10(6):725–31. 10.1123/ijspp.2015-0092 26023738

[4] Barrett S, Midgley AW, Towlson C, Garrett A, Portas M, Lovell R. Within-match playerload™ patterns during a simulated soccer match: potential implications for unit positioning and fatigue management. International journal of sports physiology and performance. 2016;11(1):135–40. 10.1123/ijspp.2014-0582 26114855

[5] Gabbett TJ, Jenkins DG. Relationship between training load and injury in professional rugby league players. Journal of Science and Medicine in Sport. 2011;14(3):204–9. 10.1016/j.jsams.2010.12.002 21256078

[6] Delaney J, Thornton H, Duthie G, Dascombe B. Factors that influence running intensity in interchange players within professional rugby league. International journal of sports physiology and performance. 2016;11(8):1047–52. 10.1123/ijspp.2015-0559 26999533

[7] Aughey RJ, Falloon C. Real-time versus post-game GPS data in team sports. Journal of Science and Medicine in Sport 2010;13(3):348–9. 10.1016/j.jsams.2009.01.006 . 19589726

[8] Larsson P. Global positioning system and sport-specific testing. Sports medicine. 2003;33(15):1093–101. 10.2165/00007256-200333150-00002 . 14719979

[9] Varley MC, Fairweather IH, Aughey RJ. Validity and reliability of GPS for measuring instantaneous velocity during acceleration, deceleration, and constant motion. Journal of sports sciences. 2012;30(2):121–7. 10.1080/02640414.2011.627941 . 22122431

[10] Di Prampero P, Fusi S, Sepulcri L, Morin J, Belli A, Antonutto G. Sprint Running: A New Energetic Approach. Journal of experimental biology. 2005;208(14):2809–16. 10.1242/jeb.01700 16000549

[11] Rawstorn JC, Maddison R, Ali A, Foskett A, Gant N. Rapid directional change degrades gps distance measurement validity during intermittent intensity running. PloS one. 2014;9(4):e93693 10.1371/journal.pone.0093693 . 24733158 PMC3986049

[12] Akenhead R, French D, Thompson KG, Hayes PR. The acceleration dependent validity and reliability of 10Hz GPS. Journal of Science and Medicine in Sport. 2014;17(5):562–6. Epub 2013/09/18. 10.1016/j.jsams.2013.08.005 . 24041579

[13] Jennings D, Cormack S, Coutts AJ, Boyd L, Aughey RJ. The validity and reliability of gps units for measuring distance in team sport specific running patterns. International journal of sports physiology and performance. 2010;5(3):328–41. 10.1123/ijspp.5.3.328 . 20861523

[14] Buchheit M, Manouvrier C, Cassirame J, Morin JB. Monitoring locomotor load in soccer: is metabolic power, powerful? International journal of sports medicine. 2015;36(14):1149–55. 10.1055/s-0035-1555927 26393813

[15] Coutts AJ, Duffield R. Validity and reliability of GPS devices for measuring movement demands of team sports. Journal of Science and Medicine in Sport 2010;13(1):133–5. 10.1016/j.jsams.2008.09.015 . 19054711

[16] Boyd LJ, Ball K, Aughey RJ. Quantifying external load in Australian football matches and training using accelerometers. International journal of sports physiology and performance. 2013;8(1):44–51. 10.1123/ijspp.8.1.44 . 22869637

[17] Howe ST, Aughey RJ, Hopkins WG, Cavanagh BP, Stewart AM, editors. Quantifying important differences in athlete movement during collision-based team sports: Accelerometers outperform Global Positioning Systems. 2017 IEEE International Symposium on Inertial Sensors and Systems (INERTIAL); 2017 2017: IEEE.

[18] Weaving D, Marshall P, Earle K, Nevill A, Abt G. Combining internal- and external-training-load measures in professional rugby league. International journal of sports physiology and performance. 2014;9(6):905–12. 10.1123/ijspp.2013-0444 . 24589469

[19] Walker EJ, McAinch AJ, Sweeting A, Aughey RJ. Inertial sensors to estimate the energy expenditure of team-sport athletes. Journal of Science and Medicine in Sport. 2015;19(2):177–81. 10.1016/j.jsams.2015.01.013 25804422

[20] Rowell AE, Aughey RJ, Hopkins WG, Stewart AM, Cormack SJ. Identification of sensitive measures of recovery following external load from football match play. International journal of sports physiology and performance. 2016;12(7):969–76. 10.1123/ijspp.2016-0522 27967334

[21] Kelly SJ, Murphy AJ, Watsford ML, Austin D, Rennie M. Reliability and validity of sports accelerometers during static and dynamic testing. International journal of sports physiology and performance. 2015;10(1):106–11. 10.1123/ijspp.2013-0408 24911138

[22] Boyd LJ, Ball K, Aughey RJ. The reliability of MinimaxX accelerometers for measuring physical activity in Australian football. International journal of sports physiology and performance. 2011;6(3):311–21. 10.1123/ijspp.6.3.311 . 21911857

[23] Hulin BT, Gabbett TJ, Johnston RD, Jenkins DG. Wearable microtechnology can accurately identify collision events during professional rugby league match-play. Journal of Science and Medicine in Sport. 2017;20(7):638–42. 10.1016/j.jsams.2016.11.006 28153609

[24] McNamara DJ, Gabbett TJ, Chapman P, Naughton G, Farhart P. The validity of microsensors to automatically detect bowling events and counts in cricket fast bowlers. International journal of sports physiology and performance. 2015;10(1):71–5. 10.1123/ijspp.2014-0062 . 24911322

[25] Cormack SJ, Mooney MG, Morgan W, McGuigan MR. Influence of neuromuscular fatigue on accelerometer load in elite australian football players. International journal of sports physiology and performance. 2013;8(4):373–8. 10.1123/ijspp.8.4.373 . 23170747

[26] Aughey RJ. Applications of GPS technologies to field sports. International journal of sports physiology and performance. 2011;6(3):295–310. 10.1123/ijspp.6.3.295 . 21911856

[27] Banister E. Modeling elite athletic performance. Physiological testing of elite athletes. 1991:403–24.

[28] Deutsch MU, Kearney GA, Rehrer NJ. Time–motion analysis of professional rugby union players during match-play. Journal of sports sciences. 2007;25(4):461–72. 10.1080/02640410600631298 17365533

[29] Aughey RJ. Australian football player work rate: evidence of fatigue and pacing. International journal of sports physiology and performance. 2010;5(3):394–405. 10.1123/ijspp.5.3.394 20861528

[30] Faude O, Koch T, Meyer T. Straight sprinting is the most frequent action in goal situations in professional football. Journal of sports sciences. 2012;30(7):625–31. 10.1080/02640414.2012.665940 22394328

[31] Gabbett T, Gahan C. Repeated high-intensity effort activity in relation to tries scored and conceded during rugby league match-play. International journal of sports physiology and performance. 2016;11(4):530–4. 10.1123/ijspp.2015-0266 26389863

[32] Varley MC, Elias GP, Aughey RJ. Current match-analysis techniques’ underestimation of intense periods of high-velocity running. International journal of sports physiology and performance. 2012;7(2):183–5. 10.1123/ijspp.7.2.183 . 22634968

[33] Cunningham DJ, Shearer DA, Carter N, Drawer S, Pollard B, Bennett M, et al Assessing worst case scenarios in movement demands derived from global positioning systems during international rugby union matches: Rolling averages versus fixed length epochs. PloS one. 2018;13(4):e0195197 10.1371/journal.pone.0195197 29621279 PMC5886488

[34] Ferraday K, Hills SP, Russell M, Smith J, Cunningham DJ, Shearer D, et al A comparison of rolling averages versus discrete time epochs for assessing the worst-case scenario locomotor demands of professional soccer match-play. Journal of Science and Medicine in Sport. 2020. 10.1016/j.jsams.2020.01.002 31937507

[35] Whitehead S, Till K, Weaving D, Jones B. The use of microtechnology to quantify the peak match demands of the football codes: a systematic review. Sports medicine. 2018:1–27. 10.1007/s40279-018-0965-6 PMC6182461 30088218

[36] Delaney JA, Thornton HR, Pryor JF, Stewart AM, Dascombe BJ, Duthie GM. Peak running intensity of international rugby: implications for training prescription. International journal of sports physiology and performance. 2016;12(8):1039–45. 10.1123/ijspp.2016-0469 27967337

[37] Delaney JA, Thornton HR, Burgess DJ, Dascombe BJ, Duthie GM. Duration-specific running intensities of Australian Football match-play. Journal of Science and Medicine in Sport. 2017;20(7):689–94. 10.1016/j.jsams.2016.11.009 28131505

[38] Delaney JA, Thornton HR, Rowell AE, Dascombe BJ, Aughey RJ, Duthie GM. Modelling the decrement in running intensity within professional soccer players. Science and Medicine in Football. 2017:1–7. 10.1080/24733938.2017 .1383623

[39] Buchheit M, Al Haddad H, Simpson BM, Palazzi D, Bourdon PC, Di Salvo V, et al Monitoring accelerations with gps in football: time to slow down? International journal of sports physiology and performance. 2014;9(3):442–5. Epub 2014/04/24. 10.1123/ijspp.2013-0187 . 23916989

[40] Akenhead R, Nassis GP. Training load and player monitoring in high-level football: current practice and perceptions. International journal of sports physiology and performance. 2016;11(5):587–93. 10.1123/ijspp.2015-0331 26456711

[41] Osgnach C, Poser S, Bernardini R, Rinaldo R, di Prampero PE. Energy cost and metabolic power in elite soccer: a new match analysis approach. Medicine & Science in Sports & Exercise. 2010;42(1):170–8. 10.1249/MSS.0b013e3181ae5cfd . 20010116

[42] Highton J, Mullen T, Norris J, Oxendale C, Twist C. Energy expenditure derived from micro-technology is not suitable for assessing internal load in collision-based activities. International journal of sports physiology and performance. 2016;12(2):264–7. 10.1123/ijspp.2016-0069 . 27193085

[43] Townshend AD, Worringham CJ, Stewart IB. Assessment of speed and position during human locomotion using nondifferential GPS. Medicine & Science in Sports & Exercise. 2008;40(1):124–32. 10.1249/mss.0b013e3181590bc2 18091013

[44] Varley MC, Jaspers A, Helsen WF, Malone JJ. Methodological considerations when quantifying high-intensity efforts in team sport using global positioning system technology. International journal of sports physiology and performance. 2017;12(8):1059–68. 10.1123/ijspp.2016-0534 28051343

[45] Hopkins WG, Marshall SW, Batterham AM, Hanin J. Progressive statistics for studies in sports medicine and exercise science. Medicine & Science in Sports & Exercise. 2009;41(1):3–12. 10.1249/MSS.0b013e31818cb278 . 19092709

[46] Hopkins WG. A spreadsheet to compare means of two groups. Sportscience. 2007;11:22–4.

[47] Smith TB, Hopkins WG. Variability and predictability of finals times of elite rowers. Medicine & Science in Sports & Exercise. 2011;43(11):2155–60. 10.1249/MSS.0b013e31821d3f8e . 21502896

[48] Hopkins WG. Spreadsheets for analysis of validity and reliability. Sportscience. 2015;19:36–42.

[49] Jones MR, West DJ, Crewther BT, Cook CJ, Kilduff LP. Quantifying positional and temporal movement patterns in professional rugby union using global positioning system. European journal of sport science. 2015;15(6):488–96. 10.1080/17461391.2015.1010106 . 25675258

[50] Roe G, Halkier M, Beggs C, Till K, Jones B. The use of accelerometers to quantify collisions and running demands of rugby union match-play. International Journal of Performance Analysis in Sport. 2016;16(2):590–601.

[51] Duthie G, Pyne D, Hooper S. Applied physiology and game analysis of rugby union. Sports medicine. 2003;33(13):973–91. Epub 2003/11/11. 10.2165/00007256-200333130-00003 . 14606925

[52] McLaren SJ, Weston M, Smith A, Cramb R, Portas MD. Variability of physical performance and player match loads in professional rugby union. Journal of Science and Medicine in Sport. 2015;19(6):493–7. 10.1016/j.jsams.2015.05.010 26118848

[53] Buchheit M, Simpson BM. Player tracking technology: half-full or half-empty glass? International journal of sports physiology and performance. 2017;12(Suppl 2):S2-35-S2-41. 10.1123/ijspp.2016-0499 27967285

[54] Buchheit M. The numbers will love you back in return—I promise. International journal of sports physiology and performance. 2016;11(4):551–4. 10.1123/IJSPP.2016-0214 27164726

[55] Quarrie KL, Hopkins WG, Anthony MJ, Gill ND. Positional demands of international rugby union: evaluation of player actions and movements. Journal of Science and Medicine in Sport. 2013;16(4):353–9. 10.1016/j.jsams.2012.08.005 22975233

[56] Lindsay A, Draper N, Lewis J, Gieseg SP, Gill N. Positional demands of professional rugby. European journal of sport science. 2015;15(6):480–7. 10.1080/17461391.2015.1025858 . 25830235

[57] Roberts SP, Trewartha G, Higgitt RJ, El-Abd J, Stokes KA. The physical demands of elite English rugby union. Journal of sports sciences. 2008;26(8):825–33. 10.1080/02640410801942122 . 18569548

[58] Lacome M, Piscione J, Hager J-P, Bourdin M. A new approach to quantifying physical demand in rugby union. Journal of sports sciences. 2013;32(3):290–300. 10.1080/02640414.2013.823225 24016296

[59] Owen SM, Venter RE, du Toit S, Kraak WJ. Acceleratory match-play demands of a Super Rugby team over a competitive season. Journal of sports sciences. 2015;33(18):2061–9. 10.1080/02640414.2015.1028086 25846204

[60] Stevens TGA, De Ruiter CJ, Van Maurik D, Wilhelmus Van Lierop CJ, Savelsbergh GJP, Beek PJ. Measured and estimated energy cost of constant and shuttle running in soccer players. Medicine & Science in Sports & Exercise. 2014;47(6):1219–24. 10.1249/MSS.0000000000000515 . 25211365

[61] Brown DM, Dwyer DB, Robertson SJ, Gastin PB. Metabolic power method underestimates energy expenditure in field sport movements using a GPS tracking system. International journal of sports physiology and performance. 2016;11(8):1067–73. Epub 2016/03/22. 10.1123/ijspp.2016-0021 . 26999381

[62] Mohr M, Krustrup P, Bangsbo J. Fatigue in soccer: a brief review. Journal of sports sciences. 2005;23(6):593–9. 10.1080/02640410400021286 16195008

[63] Waldron M, Highton J. Fatigue and pacing in high-intensity intermittent team sport: an update. Sports medicine. 2014;44(12):1645–58. 10.1007/s40279-014-0230-6 . 25047854

[64] Vanrenterghem J, Nedergaard NJ, Robinson MA, Drust B. Training load monitoring in team sports: a novel framework separating physiological and biomechanical load-adaptation pathways. Sports medicine. 2017;47(11):2135–42. Epub Mar 10. 10.1007/s40279-017-0714-2 28283992

[65] Boyd L, Gallaher E, Ball K, Stepto N, Aughey R, Varley M. Practical application of accelerometers in Australian football. Journal of Science and Medicine in Sport. 2010;13:e14–e5. 10.1016/j.jsams.2010.10.491

[66] Barrett S, Midgley A, Lovell R. Playerload™: reliability, convergent validity, and influence of unit position during treadmill running. International journal of sports physiology and performance. 2014;9(6):945–52. 10.1123/ijspp.2013-0418 . 24622625

[67] McLellan CP, Coad S, Marsh D, Lieschke M. Performance analysis of super 15 rugby match-play using portable micro-technology. J Athl Enhancement. 2013;2(5). 10.4172/2324-9080.1000126

